# Success rate of acceleromyographic neuromuscular monitoring based on the depth of anesthesia at the time of sugammadex antagonism: a randomized controlled trial

**DOI:** 10.1177/03000605241305474

**Published:** 2024-12-15

**Authors:** Hwang-Ju You, Seok-Jin Lee, Ji-Yoon Jung, Sung-Ae Cho, Woojin Kwon, Jin-Bum Kim, Tae-Yun Sung

**Affiliations:** 1 Department of Anaesthesiology and Pain Medicine, 384997 Konyang University Hospital, Konyang University College of Medicine, Daejeon, Korea; 2 Department of Anaesthesiology and Pain Medicine, 384997 Konyang University Hospital, Konyang University Myunggok Medical Research Institute, Konyang University College of Medicine, Daejeon, Korea; 3 Department of Urology, 384997 Konyang University Hospital, Konyang University College of Medicine, Daejeon, Korea

**Keywords:** Sugammadex, neuromuscular monitoring, consciousness monitor, train-of-four, emergence agitation, anesthesia, acceleromyography, bispectral index

## Abstract

**Objective:**

We aimed to compare the success of non-normalized acceleromyographic neuromuscular monitoring and recovery profiles based on the depth of anesthesia at the time of sugammadex administration.

**Methods:**

Patients undergoing general anesthesia were prospectively and randomly allocated to two groups. In the BIS60 group, sugammadex was administered when there was a bispectral index (BIS) <60 and anesthesia was maintained until a train-of-four ratio ≥1.0 was obtained, whereas in the BIS70 group, anesthesia was stopped at the completion of surgery, sugammadex was administered when the BIS was >70, and the recovery of neuromuscular function was monitored. The recovery profile and the incidence of emergence agitation of the two groups were compared.

**Results:**

The success rate of neuromuscular monitoring was significantly higher for the BIS60 group than for the BIS70 group (100% *vs*. 37.5%, respectively). The time taken for recovery for the two groups was comparable. The incidence of emergence agitation was significantly lower in the BIS60 group than in the BIS70 group (23.3% *vs*. 56.3%, respectively).

**Conclusion:**

After the administration of sugammadex, the maintenance of anesthesia until the full recovery of neuromuscular function increases the success rate of neuromuscular monitoring without delaying recovery and reduces the risk of emergence agitation.

**Clinical trial registration:** CRIS registration number KCT0007899 (https://cris.nih.go.kr/)

## Introduction

To prevent residual neuromuscular blockade owing to the use of neuromuscular blocking agents (NMBAs) in patients undergoing general anesthesia, the confirmation of neuromuscular recovery before extubation by means of quantitative neuromuscular monitoring (NMM) is strongly recommended.^1,2^ However, all NMM methods are sensitive to patient movement to various extents,^3^ and a failure of monitoring commonly occurs secondary to involuntary and/or non-purposeful movements of the patient during their emergence from general anesthesia.

Inhalation anesthetics that are used for general anesthesia cause muscle relaxation and potentiate the effects of NMBAs. Therefore, most anesthesiologists administer neuromuscular blockade reversal agents after discontinuing the administration of inhalation anesthetics.^4,5^ However, the optimal timing of sugammadex administration remains unclear. A previous study showed that sugammadex administration is associated with a higher risk of upper airway obstruction when the minimum alveolar concentration of the inhalation anesthetic is ≥0.3.^4^ However, the administration of sugammadex is associated with an increase in the bispectral index (BIS) and clinical signs of awakening. Therefore, it has been recommended to maintain anesthesia until a full recovery of neuromuscular function is achieved to prevent potential episodes of awareness.^6^ In addition, patient agitation during emergence from anesthesia often precludes adequate NMM.

Sugammadex, a selective antagonist of aminosteroidal NMBAs, reverses neuromuscular blockade within a mean of 2 to 3 minutes when it is administered at an appropriate dose. The amount of time required depends on the level of neuromuscular blockade, and the effectiveness and speed of reversal are dose-dependent.^7^ The recommended dose of sugammadex for the reversal of neuromuscular blockade according to the level of neuromuscular blockade is well known, but the timing of sugammadex administration varies between anesthetists. Moreover, there have been few studies of the appropriate timing of sugammadex administration and its effects on recovery profiles.^8^

The authors hypothesized that the administration of sugammadex during the maintenance phase of anesthesia would increase the success rate of quantitative NMM and reduce the risk of emergence agitation, without affecting the length of the period between the end of surgery and extubation. Therefore, in the present study, we compared the success rates of quantitative NMM and the recovery profiles of patients who were administered sugammadex at different times.

## Materials and methods

### Study sample

We performed a prospective, randomized, controlled study that was approved by the Institutional Review Board of Konyang University Hospital, Daejeon, Republic of Korea (approval number: KYUH 2022-08-039-002, approval date: 11 October 2022) and registered with the Korean Clinical Research Information Service (https://cris.nih.go.kr/, permit number: KCT0007899) before patient enrollment. Written informed consent was obtained from all the participants. The reporting of this study conforms to the CONSORT statements.^9^ The study was conducted in accordance with the principles of the Declaration of Helsinki of 1975, as revised in 2013.

We enrolled patients aged 20 to 65 years who had an American Society of Anesthesiologists (ASA) physical status of I to II and were scheduled to undergo elective facial trauma surgery under general anesthesia between December 2022 and November 2023 at a single university hospital. The exclusion criteria were any contraindications to sugammadex administration, such as hypersensitivity/anaphylactic reaction to sugammadex and renal failure with an estimated glomerular filtration rate of <30 mL/minute; a body mass index of >30 kg/m^2^; and the presence of a neuromuscular disorder, moderate or severe liver, kidney, heart, or respiratory disease, or cognitive impairment. In addition, patients with factors that could have affected their postoperative recovery profile, such as those undergoing a combination of surgical procedures, were excluded.

Patients were randomly assigned to one of two groups: one in which patients were administered sugammadex when they had a BIS of <60 (BIS60 group) and another in which patients were administered the drug when they had a BIS of >70 (BIS70 group). The patients were allocated to these groups at a 1:1 ratio by an assistant who was not otherwise involved in the study using online randomization software (Researcher Randomizer; Social Psychology Network, Evansville, IN, USA). The group allocation was concealed using a sealed opaque envelope, and was revealed 10 minutes before the anticipated end of the surgical procedure by the attending anesthesiologist, who administered the sugammadex.

### Anesthesia

The patients entered the operating room without having been administered premedication, and standard monitoring, including pulse oximetry, non-invasive blood pressure (BP) monitoring, electrocardiography, and bispectral index (BIS VISTA™ monitor; Aspect Medical Systems, Norwood, MA, USA) monitoring, was conducted. NMM was performed using an accelerometer (IntelliVue; Philips, Amsterdam, Netherlands) that was placed on the adductor pollicis muscle of the arm in which blood pressure monitoring was not being performed.

Following preoxygenation via a facial mask by means of tidal volume breathing for 3 minutes using 6 L/minute of 100% oxygen, anesthesia was induced using 2 mg/kg propofol and 1 μg/kg fentanyl. After loss of consciousness, the acceleromyograph was calibrated automatically using its built-in protocol, and the supramaximal stimulation level was determined. Train-of-four (TOF) stimulation (supramaximal stimuli, 0.2-ms duration; frequency, 2 Hz) was then started. After stabilization (i.e. when there was <5% variability in the amplitude of the response to nerve stimulation over ≥2 minutes) of the neuromuscular measurements and the establishment of baseline values, 0.6 mg/kg rocuronium was administered. Endotracheal intubation was performed when the TOF count was 0, and volume-controlled mechanical ventilation was initiated. The TOF ratio was measured every 12 s until intubation, every 5 minutes until the end of the surgery, and every 12 s after sugammadex administration until extubation. The parameters that were initially used for mechanical ventilation were a tidal volume of 7 to 8 mL/kg and a respiratory rate of 12 breaths/minute, and these were adjusted to maintain an end-tidal carbon dioxide partial pressure (EtCO_2_) of 30 to 40 mmHg during anesthesia. Anesthesia was maintained using an O_2_/50% N_2_O mixture (fraction of inspired O_2_ concentration [FiO2], 0.5), and the end-tidal concentration of desflurane was adjusted to achieve a target BIS of 40 to 60. During surgery, rocuronium was administered intermittently at a dose of 0.1 to 0.2 mg/kg to achieve moderate neuromuscular blockade (TOF count 1 to 3) at the end of the surgery.^1–3^

The surgical drapes were removed upon completion of the surgery. This was followed by the insertion of an oral airway device, and suctioning of the oral cavity was performed for both groups. In addition, we ensured that the movement of the thumb to which the NMM device was applied was unimpeded. Subsequently, sugammadex was administered, and the inhalation anesthetic was discontinued as follows. For the BIS60 group, sugammadex was administered when the BIS was <60, and the BIS was maintained at <60 using the inhalation anesthetic (50% N_2_O ± 2 vol% of desflurane) until a TOF ratio of ≥1.0 was obtained, when the administration of the inhalation anesthetic was discontinued. For the BIS70 group, the administration of the inhalation anesthetics was first discontinued, the BIS was confirmed to be >70, and sugammadex was then administered. In both groups, sugammadex (2 mg/kg) was intravenously injected to reverse the neuromuscular blockade based on NMM. After the discontinuation of inhalation anesthetic administration, the patients were ventilated with 100% O_2_ at 6 L/minute and extubated according to the same extubation criteria, which were a spontaneous tidal volume >300 mL, a respiratory rate of 8 to 20 breaths/minute, and an EtCO_2_ of ≤45 mmHg).^5^

### Assessments made

The duration of anesthesia (from the administration of the induction drug to the discontinuation of the inhalation anesthetic); the duration of surgery (from the beginning of the surgical intervention [e.g., nasal packing or skin incision] to the end of the surgical intervention [e.g., completion of the surgical dressing]); the time taken to recover (from the completion of surgery to extubation); the duration of the period from the discontinuation of the inhalation anesthetic to the recovery of spontaneous breathing, the response to verbal commands (“open your eyes,” “squeeze my hand”), extubation, and name recall; and the maximum TOF ratio before extubation were recorded. The severity of agitation and coughing was assessed during the emergence period, which was defined as the period from the discontinuation of the inhalation anesthetic to 3 minutes after extubation. Agitation was assessed using the Ricker Sedation–Agitation Scale (RSAS; scores: 1, unarousable; 2, highly sedated; 3, sedated; 4, calm and cooperative; 5, agitated but responding calmly to verbal instruction; 6, very agitated, requiring restraint; 7, pulling at the tracheal tube, trying to remove catheters, or striking the staff).^8^ An RSAS score ≥5 was defined as indicating emergence agitation. Coughing was assessed on a four-point scale (0, no cough; 1, mild, single cough; 2, moderate, more than one cough, lasting for <5 s; 3, severe, sustained coughing for ≥5 s),^10^ and a score of ≥1 was taken to indicate the presence of coughing. Airway obstruction was defined using the requirement for continuous (>30 s) positive pressure ventilation, with or without an airway device in place, and its occurrence was recorded.

All the patients were transferred to the post-anesthesia care unit (PACU) and observed for at least 40 minutes beginning at 3 to 5 minutes following extubation. During their stay in the PACU, the patients were assessed for postoperative pain using a numeric rating scale (NRS; 0, no pain; 10, worst pain imaginable), and 0.5 to 1 μg/kg fentanyl was injected intravenously if the NRS score was >4. All adverse events were recorded.

The attending anesthesiologist could not be blinded because of the differing timing of sugammadex administration but was not involved in the evaluation of the outcomes or data collection. Patient evaluation and data collection were conducted by an anesthesiology resident who was unaware of the purpose of the study and the group allocation of the patients.

The collection of three consecutive TOF ratios ≥1.0 during recovery was defined as indicating successful neuromuscular function monitoring, and the success rates of the two groups were compared as the primary outcome of the study. The secondary outcomes were the frequency of a TOF ratio ≥0.9 during recovery; the time taken to recover; the duration from the discontinuation of the inhalation anesthetic to the recovery of spontaneous breathing, the response to verbal commands, and extubation; and the occurrence of emergence agitation; the occurrence and severity of coughing; the occurrence of airway obstruction; and the occurrence of adverse events.

### Statistical analyses

The required sample size was calculated using the assumption that the difference in the success rate of neuromuscular function monitoring would be >30% (BIS60 group *vs*. BIS70 group: 100% *vs*. 70%, respectively). Using a Cohen’s h effect size of 1.159, an α-value of 0.05 (two-tailed), a power of 0.9, and an allocation ratio of 1:1, 28 patients per group were calculated to be required to demonstrate a difference of this size. When an estimated dropout rate of 10% was added, a target of 32 patients per group was used.

IBM SPSS Statistics software ver. 27.0 (IBM Corp., Armonk, NY, USA) was used for the statistical analyses. Continuous datasets were compared using Student’s *t*-test or the Mann–Whitney U test, depending on the results of Kolmogorov–Smirnov normality testing. Categorical datasets were compared using the χ^2^ test, the χ^2^ test for trends (linear-by-linear association), or Fisher’s exact test, as appropriate. Statistical significance was accepted when *p* < 0.05. Cohen’s d and h effect sizes were used to compare the continuous and categorical datasets, respectively.

## Results

**Figure 1.**
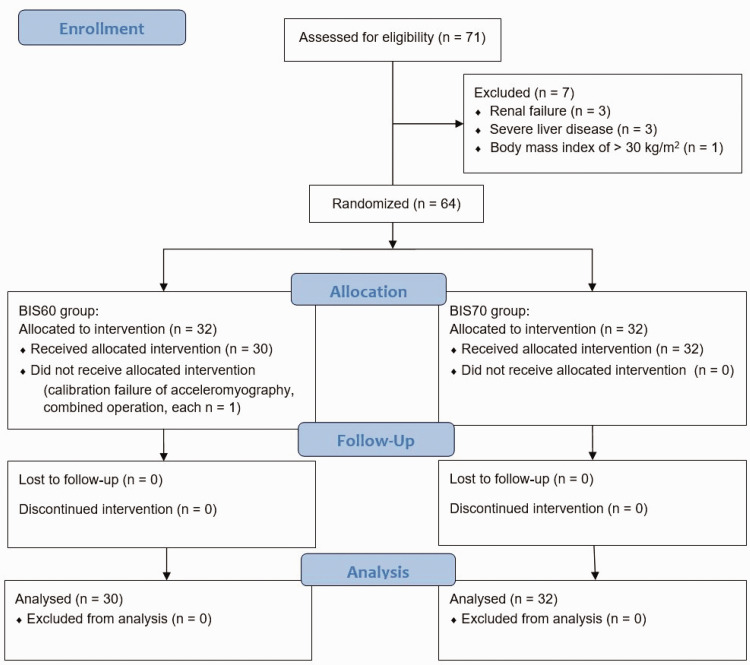
CONSORT participant flow chart.

Of the 71 eligible patients, 7 were excluded (3 had renal failure, 3 had severe liver disease, and 1 had a body mass index of >30 kg/m^2^. Therefore, 64 remained and were randomly assigned to the two groups. In the BIS60 group, a further two patients were excluded because of a failure of calibration of the acceleromyograph and a combination of surgical procedures. Consequently, 30 and 32 patients were studied in the BIS60 and BIS70 groups, respectively (Figure 1). The characteristics of and perioperative data for the patients in the two groups were comparable (Table 1). The success rates of NMM and the recovery profiles of the patients in each group are presented in Table 2.

**Table 1.** Patient characteristics and perioperative data.

Variable	BIS60 group(n = 30)	BIS70 group(n = 32)	*P*
Age (years)	40.6 ± 11.6	40.7 ± 14.0	0.963
Sex (male/female)	23/7	23/9	0.667
Height (cm)	167.5 ± 7.0	168.6 ± 7.6	0.544
Body mass (kg)	71.2 ± 11.1	68.6 ± 10.4	0.332
Body mass index (kg/m^2^)	25.3 ± 2.6	24.0 ± 2.6	0.060
ASA physical status I/II	7/28	9/23	0.667
Surgical site			0.271
Orbital wall	16 (53.3%)	14 (43.8%)	0.450
Nose	10 (33.3%)	9 (28.1%)	0.657
Zygomatic/maxillary	3 (10.0%)	7 (21.9%)	0.304
Zygoma	1 (3.3%)	2 (6.3%)	>0.999
Baseline TOF ratio	112.9 ± 10.1	115.8 ± 9.4	0.236
Supramaximal stimulation (mA)	55.0 (50.0–60.0)	60.0 (47.5–60.0)	0.520
Fluids administered (mL)	200.0 (150.0–400.0)	250.0 (150.0–400.0)	0.701
Duration of surgery (min)	59.5 (12.8–95.2)	66.5 (15.2–131.5)	0.334
Duration of anesthesia (min)	77.6 ± 51.4	92.2 ± 60.9	0.312

Values are mean ± standard deviation (SD), number of patients, number (%), or median (interquartile range). Continuous datasets were compared using Student’s *t*-test or the Mann–Whitney U test, as appropriate. Categorical datasets were compared using the χ^2^ test, χ^2^ test for trends (linear-by-linear association), or Fisher’s exact test, as appropriate. ASA: American Society of Anesthesiologists; TOF: train-of-four.

**Table 2.** Success rates of neuromuscular monitoring and the recovery profiles of the patients.

	BIS60 group(n = 30)	BIS70 group(n = 32)	MD or RR (95% CI)	Effect size *h* or *d*	*P*
During recovery time					
Maximal TOF ratio (%)	112.5 (105.8–124.3)	97.0 (72.0–112.0)*	NA	NA	<0.001
Success of neuromuscular monitoring (i.e. TOF ratio ≥1.0)	30 (100%)	12 (37.5%)	62.5% (41.9%, 77.1%)	1.823	<0.001
TOF ratio ≥0.9	30 (100%)	17 (53.1%)	46.9% (27.3%, 63.6%)	1.509	<0.001
Time to recovery (min)	7.0 (6.0–8.0)	7.0 (6.0–8.8)	NA	NA	0.693
Time from the discontinuation of the inhalation anesthetic to …					
Spontaneous breathing (min)	2.9 ± 1.4	4.4 ± 2.4	−1.5 (−2.4, −0.47)	0.763	0.004
Response to verbal commands (min)	4.4 ± 1.3	6.1 ± 2.1	−1.7 (−2.5, −0.8)	0.973	<0.001
Extubation (min)	4.2 (3.9–4.8)	6.0 (5.2–7.0)	NA	NA	<0.001
Name recall (min)	5.4 (4.6–6.2)	7.0 (5.8–8.1)	NA	NA	<0.001
Emergence agitation (i.e., RSAS ≥5)	7 (23.3%)	18 (56.3%)	0.45 (0.23, 0.89)	0.690	0.008
RSAS (4/5/6/7)	23/4/2/1	14/6/7/5	NA	NA	0.006
Coughing	21 (70.0%)	26 (81.3%)	NA	NA	0.301
Severity of cough (0/1/2/3)	9/9/7/5	6/11/10/5	NA	NA	0.512
Airway obstruction	0	0	NA	NA	>0.999

Values are median (interquartile range), mean ± standard deviation, number (%), or number. Datasets were compared using the Mann–Whitney U test, Student’s *t*-test, the χ^2^ test, the χ^2^ test for trends, or Fisher’s exact test, as appropriate. MD, mean difference; RR, relative risk; CI, confidence interval; recovery time, time from the completion of surgery to extubation; TOF, train-of-four; NA, not applicable; RSAS, Ricker Sedation–Agitation Scale. *n = 29.

During recovery, the TOF ratios of three patients in the BIS70 group could not be measured because of movements accompanying their emergence agitation. Therefore, the maximal TOF ratio was calculated for 30 patients in the BIS60 group and 29 patients in the BIS70 group. It was significantly higher for the BIS60 group than for the BIS70 group (median [interquartile range], 112.5% [105.8%–124.3%] *vs*. 97.0% [72.0%–112.0%], *P* < 0.001; Table 2). The success rate of NMM was significantly higher for the BIS60 group than for the BIS70 group (100% [30/30] *vs*. 37.5% [12/32], respectively; mean difference [MD], 62.5%; 95% confidence interval [CI], 41.9%–77.1%; effect size d, 1.823; *P* < 0.001). The number of patients with TOF ratios ≥0.9 was also higher for the BIS60 group than for the BIS70 group (100% [30/30] *vs*. 53.1% [17/32], respectively; MD 46.9%; 95% CI, 27.3%–63.6%; effect size d, 1.509; *P* < 0.001; Table 2).

The time taken to recover for the two groups did not differ. However, the period from the discontinuation of the administration of the anesthetic to the recovery of spontaneous breathing (2.9 ± 1.4 *vs*. 4.4 ± 2.4 minutes; *P* = 0.004), the time taken to recover a response to verbal commands (4.4 ± 1.3 *vs*. 6.1 ± 2.1 minutes; *P* < 0.001), the time to extubation (4.2 [3.9–4.8] *vs*. 6.0 [5.2–7.0] minutes; *P* < 0.001), and time taken for name recall to return (5.4 [4.6–6.2] *vs*. 7.0 [5.8–8.1] minutes; *P* < 0.001) were all significantly shorter for the BIS60 group than for the BIS70 group.

Emergence agitation occurred less frequently in the BIS60 group than in the BIS70 group (23.3% [7/30] *vs*. 56.3% [18/32], respectively; relative risk 0.45; 95% CI 0.23–0.89; effect size d, 0.690; *P* = 0.008). The emergence agitation was significantly more severe in the BIS70 group than in the BIS60 group (*P* = 0.006) but the incidence and severity of coughing and incidence of airway obstruction did not differ between the groups.

In the PACU, the NRS scores for pain, the number of patients who were administered fentanyl, and the incidence of adverse events were similar for the two groups (Table 3).

**Table 3.** Postoperative pain score and adverse events encountered in the post-anesthesia care unit.

Variable	BIS60 group(n = 30)	BIS70 group(n = 32)	*P*
NRS for pain	4.0 (1.8–6.0)	3.0 (2.0–5.8)	0.690
Fentanyl	12 (40.0%)	10 (31.3%)	0.472
Adverse events			
Nausea	3 (10.0%)	3 (9.4%)	>0.999
Dry mouth	2 (6.5%)	1 (3.1%)	0.613
Dizziness	1 (3.3%)	2 (6.3%)	>0.999
Headache	0	1 (3.1%)	>0.999
Sore throat	0	1 (3.1%)	>0.999
Feeling of suffocation	1 (3.3%)	0	0.484
Shivering	1 (3.3%)	0	0.484

Values are median (interquartile range) or number (%). Datasets were compared using the Mann–Whitney U test, the χ^2^ test, or Fisher’s exact test, as appropriate. NRS, numerical rating scale (0, no pain, 10, worst pain imaginable).

## Discussion

Compared with administering sugammadex while a patient is recovering consciousness (BIS >70) after the termination of inhalation anesthesia, administering this drug while maintaining anesthesia (BIS <60) significantly increased the success rate of NMM before extubation, without delaying recovery. Furthermore, the discontinuation of the administration of anesthetics after the confirmation of the full recovery of neuromuscular function reduced the incidence and severity of emergence agitation, without increasing the incidences of coughing or airway obstruction.

TOF ratios of ≥0.9 are recommended to protect the airway from aspiration before extubation.^1,2^ However, the baseline TOF ratio measured using an acceleromyograph (AMG), the most common clinical NMM device, before neuromuscular blocker administration is higher than that measured using electromyography (EMG) or mechanomyography (MMG) and is often >1.0.^11,12^ The higher baseline TOF ratio measured using an AMG indicates that the TOF ratio required to exclude residual paralysis is higher. To prevent the overestimation of neuromuscular recovery when using an AMG, a “normalization” process is necessary, involving the use of the displayed TOF ratio at recovery/baseline TOF ratio.^12,13^ However, the application of this normalization to routine anesthesia is somewhat cumbersome, and an NMM device that provides an automatically calculated “normalized” TOF ratio is not yet available. Therefore, the use of a non-normalized AMG TOF ratio of ≥1.0 is recommended as the threshold to exclude residual paralysis.^1,2^ Considering this, we have presented the success of NMM using a non-normalized AMG TOF ratio of ≥1.0 and used this as the primary outcome.

Residual paralysis is associated with pharyngeal dysfunction, a poor ventilatory response to hypoxia, hypoxemia, upper airway obstruction, and atelectasis.^14^ Sugammadex is a reversal agent for aminosteroidal NMBA-induced neuromuscular blockade that significantly reduces the incidence of residual paralysis *vs*. conventional anticholinergic/cholinesterase inhibitor mixtures. However, the use of sugammadex does not obviate the need for quantitative monitoring.^15^ In clinical settings where sugammadex reversal without quantitative monitoring is performed, the incidences of post-extubation TOF values <0.9 and <1.0 can be as high as 9.4% and 55.6%, respectively.^16^ Considering that the incidences in the present study were calculated on the basis of uncalibrated and non-normalized AMG values, the incidences of post-extubation TOF values of <0.9 and <1.0 would be higher if EMG, MMG, or normalized AMG values were used. Moreover, residual paralysis occurred in 10% of the participants, even when the recommended dose of sugammadex was used, according to the results of peripheral nerve stimulation using a qualitative monitor after the surgery.^17^ Therefore, confirmation of the adequate recovery of neuromuscular function by means of quantitative NMM before extubation is essential and is the only suitable method for the prevention of residual paralysis.^14,15^

The guidelines of the ASA and European Society of Anaesthesiology and Intensive Care for the monitoring and antagonism of neuromuscular blockade that were published in 2023 recommend the use of sugammadex for the reversal of deep, moderate, and shallow depths of neuromuscular blockade induced by aminosteroidal NMBAs, and that the recovery of neuromuscular function should be confirmed using quantitative NMM before extubation to prevent residual paralysis.^1,2^ Nevertheless, the use of NMM in patients who have been administered NMBAs does not exceed 60%, according to the results of previous observational studies and surveys, and the recovery of neuromuscular function is only confirmed using quantitative NMM before extubation in only 16.5% of patients.^18,19^ Fluctuating TOF values and error messages generated by monitoring devices because of a failure of immobilization makes NMM difficult during emergence from anesthesia.^18^ In the present study, the higher success rate of NMM for the BIS60 group than for the BIS70 group may be attributable to the minimization of involuntary movements of the patients by maintaining anesthesia until neuromuscular function had fully recovered.

In the BIS60 group, the incidence and severity of emergence agitation were lower than in the BIS70 group. This may be attributable to the use of N_2_O to maintain a BIS value <60 from the moment a TOF ratio of ≥1.0 was confirmed after sugammadex administration. In some previous studies, N_2_O was shown to reduce the incidence of emergence agitation.^20^ In addition, incomplete recovery of neuromuscular function during the recovery of consciousness by patients in the BIS70 group may have further worsened their emergence agitation.^21^

In previous studies,^4,10^ the median timing of sugammadex administration was 2 or 6 minutes after the discontinuation of anesthesia. The mean period from the administration of sugammadex to extubation was 6.5 to 15 minutes,^10,22^ and the mean period from the discontinuation of anesthesia to extubation was 7.4 minutes.^21^ The time taken for recovery has been defined differently in previous studies and is affected by the characteristics of the patients, the type of surgery performed, and the type of anesthetic used. Therefore, it is difficult to compare data collected in various studies. In the present study, the time taken for recovery by patients in both groups was comparable; a mean of 7 minutes was recorded for both groups. The discontinuation of anesthetic administration after the recovery of neuromuscular function in the BIS60 group may have reduced the time taken for the recovery of spontaneous breathing and the response to verbal commands, and the time to extubation, although anesthesia was maintained longer after the end of surgery to confirm the recovery of neuromuscular function in the BIS60 group than in the BIS70 group.

The study had some limitations. First, AMG was used for NMM. However, a preload device (hand adapter) that was recommended by the manufacturers was not used,^23^ which may have reduced the reliability of the TOF measurements.^3^ However, the primary and secondary outcomes of the study were the success rate of NMM before extubation and the recovery profile of the patients, and therefore, this would not have had a significant effect on the results. Second, a non-normalized AMG-measured TOF ratio of ≥1.0 was defined as successful NMM. However, a non-normalized TOF ratio of ≥1.0 is not directly equivalent to a normalized TOF ratio of ≥0.9. For example, if the baseline TOF ratio is 1.13, a non-normalized TOF ratio of 1.0 measured before extubation is equivalent to a normalized TOF ratio of 0.88 (=1.0/1.13), and a normalized TOF ratio <0.9 may result in clinically significant residual paralysis.^24^ Third, we analyzed a single-center cohort with a relatively small sample size, which impedes the generalizability of the findings owing to the heterogeneity in the perioperative management of patients between centers and countries. Fourth, if a patient recovers consciousness but the effect of the residual neuromuscular blocking agent has not been reversed, the patient may feel discomfort, and the quality of recovery may be lower. Although a feeling of suffocation developed in one patient in the present study, this was probably because of the use of intranasal gauze packing after nasal surgery, not the intervention used in the study. If a study with a similar purpose is planned in the future, it would be helpful to use a tool such as the Quality of Recovery-15 questionnaire.

In conclusion, the administration of sugammadex and confirmation of a TOF ratio of ≥1.0, when the BIS is <60, increases the success rate of quantitative NMM and reduces emergence agitation without causing a delay in recovery.
